# Supplementary material for: ESPressoscope: A small and powerful approach for in situ microscopy
Source: PLoS One. 2024 Oct 16;19(10):e0306654. doi: 10.1371/journal.pone.0306654 (PMC11482665; doi:10.1371/journal.pone.0306654)

Illumination:
- Periscope
- Baloon LED
- USB LED
- Neopixel LED
- Inline Holographic LED
-Spectro-photometer

Focus:
- Magnetic
- Spring
- Automatic

Boards:
- ESP32 CAM
- ESP32S3 Xiao Sense

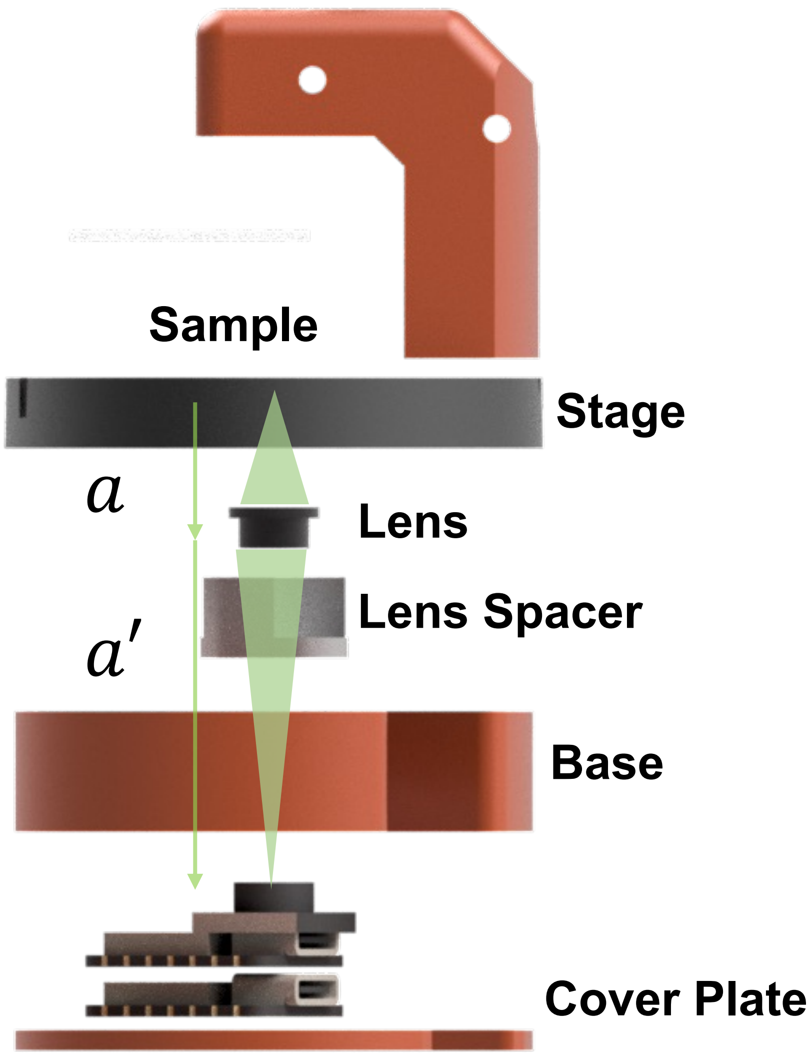

Supplement: S1 Fig — The unscrewed photo objective lens serves as the microscope imaging lens. An in-focus image is formed when 1f′=1a-1a′. The various elements of the device, such as the stage or the illumination, can be exchanged and combined depending on the specific imaging application. (PDF) [file pone.0306654.s001.pdf]
