# Supplementary figures and images for: ESPressoscope: A small and powerful approach for in situ microscopy
Source: PLoS One. 2024 Oct 16;19(10):e0306654. doi: 10.1371/journal.pone.0306654 (PMC11482665; doi:10.1371/journal.pone.0306654)

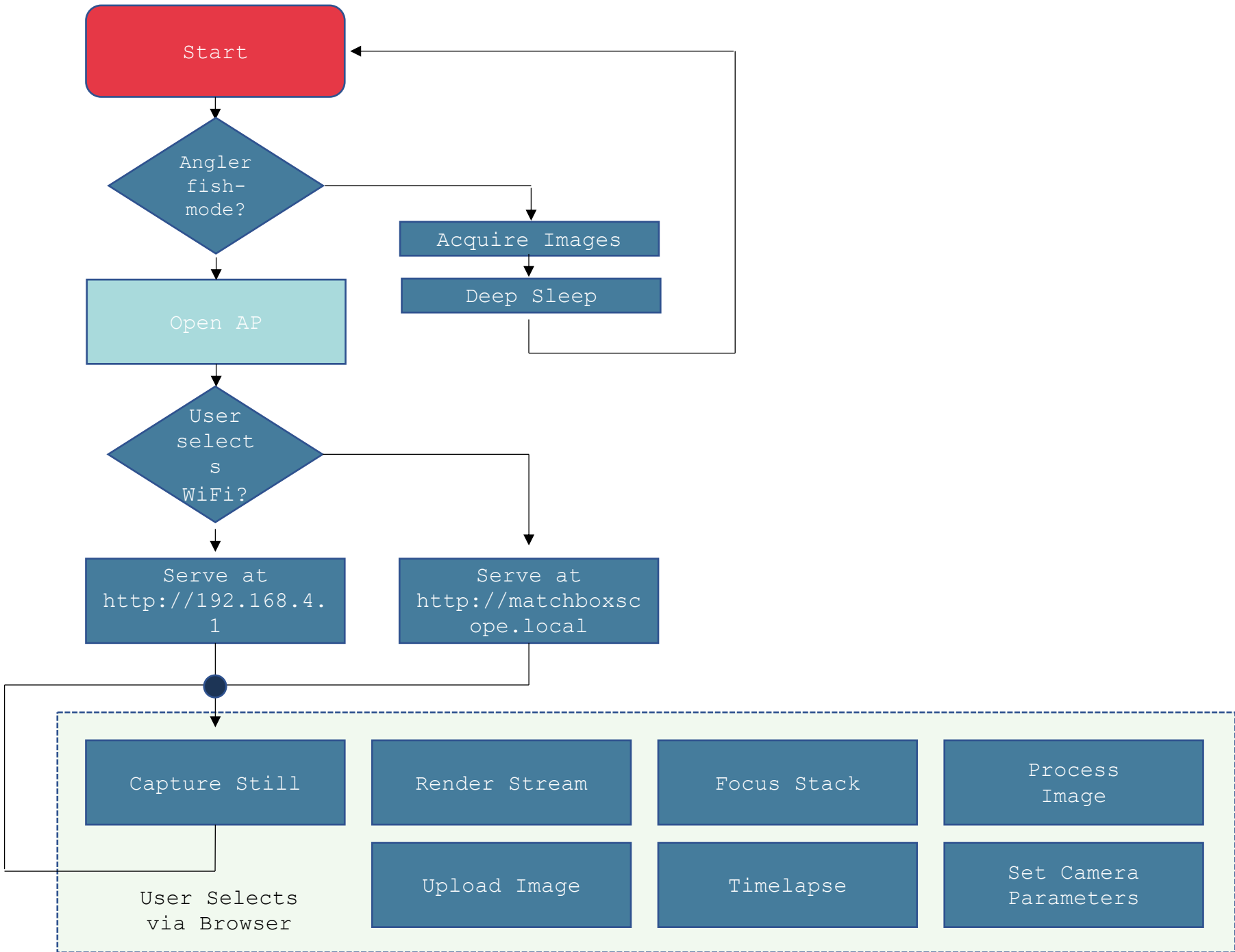

Supplement: S2 Fig — The flowchart shows the operation of the firmware, where the “Anglerfish-mode” is for autonomous operation, allowing the device to take images periodically and go into the ESP32 microcontroller’s deep sleep mode to reduce energy consumption. Alternatively, the user can control the microscope from the browser, observe its camera stream, and perform image processing tasks. (PDF) [file pone.0306654.s002.pdf]

Mean Intensity and Drift over Time

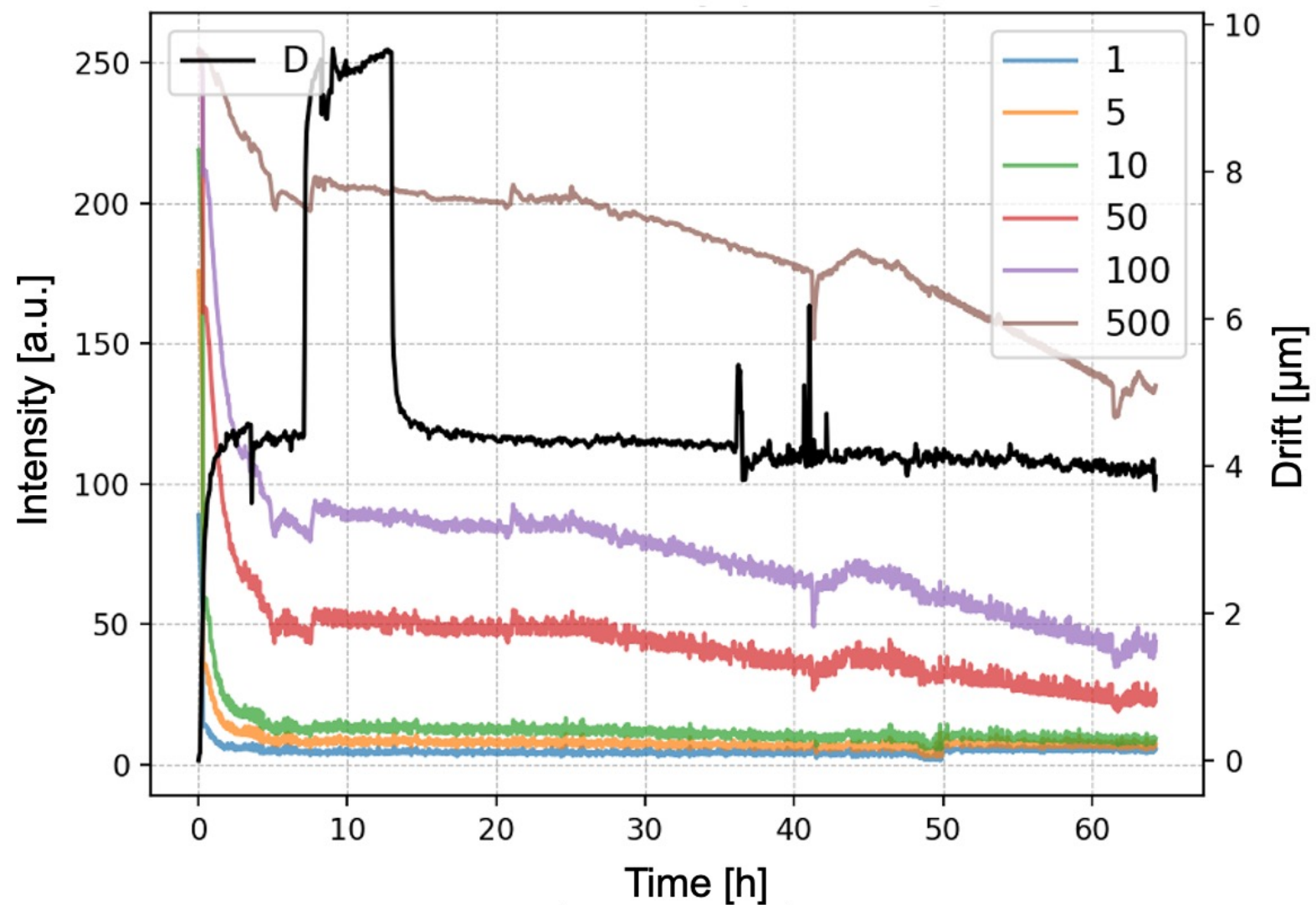

Supplement: S4 Fig — The graph shows the average intensity of images taken every minute and stored on the SD card with different exposure times (1 ms, 5 ms, 10 ms, 50 ms, 100 ms, and 500 ms). The microscope was placed in a dark box with the external battery-powered LED light switched on. Even after 3 days of constant illumination, the image intensity at longer exposure times is sufficient for brightfield microscopy. Lateral drift of the time series, calculated as a cross-correlation of successive images, shows a sudden jump in value after about 8 hours, likely due to the opening of a door. (PDF) [file pone.0306654.s004.pdf]

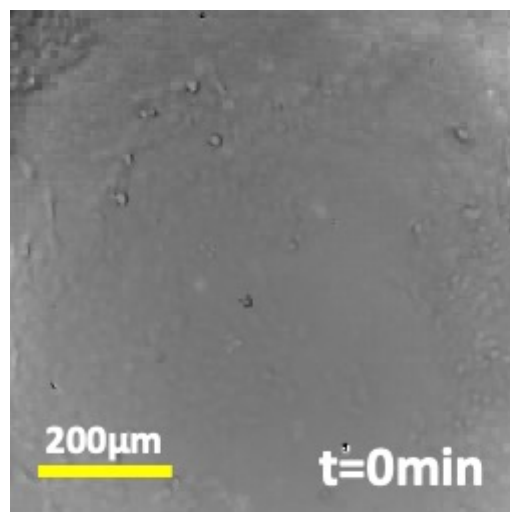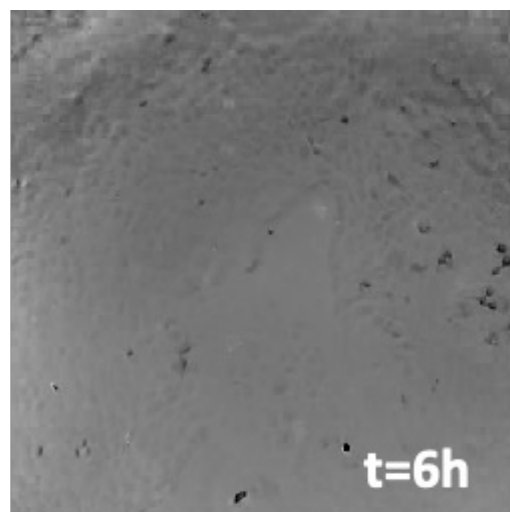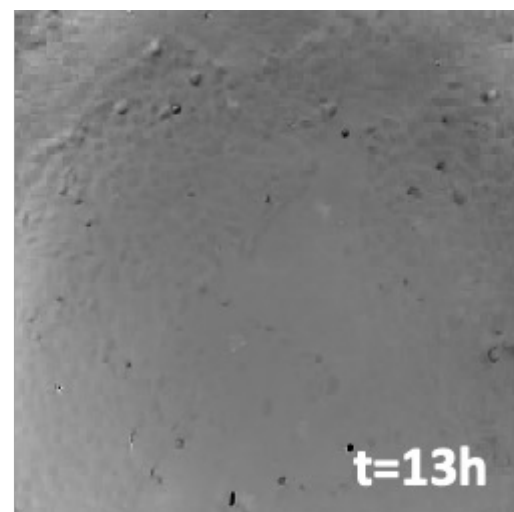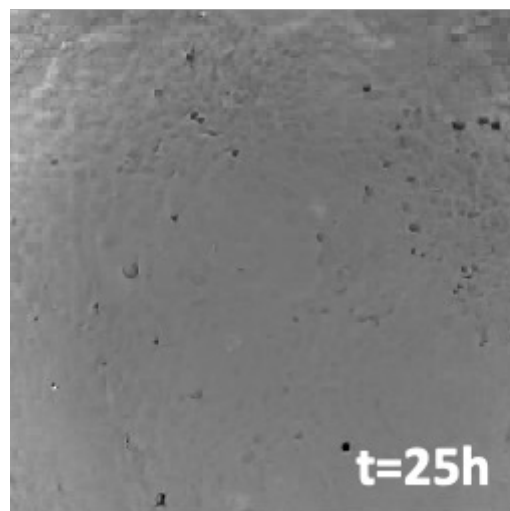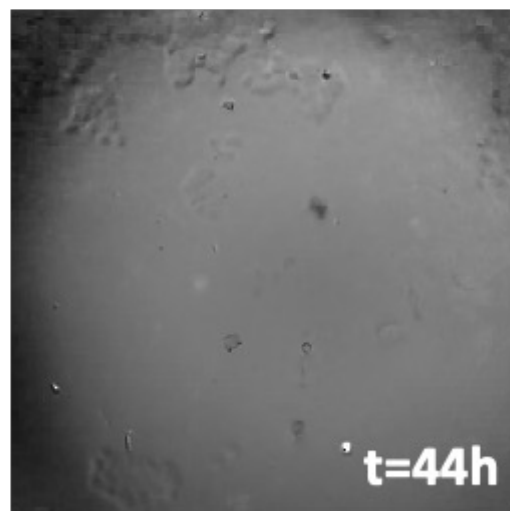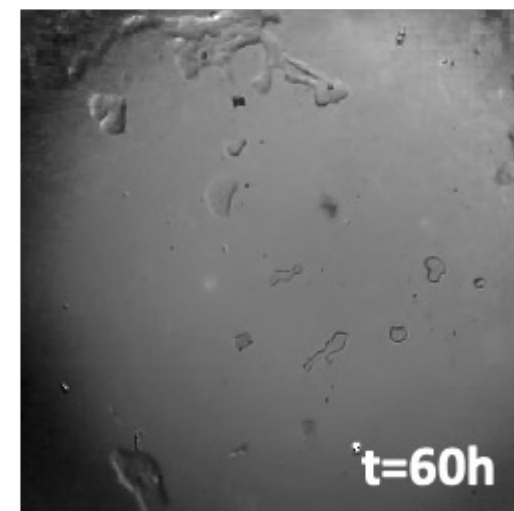

Supplement: S5 Fig — HeLa cells in a standard 25mm petri dish are shown. After around 2 days, the cells stopped replicating, most likely due to the increased temperature of the ESP32 camera module. (PDF) [file pone.0306654.s005.pdf]

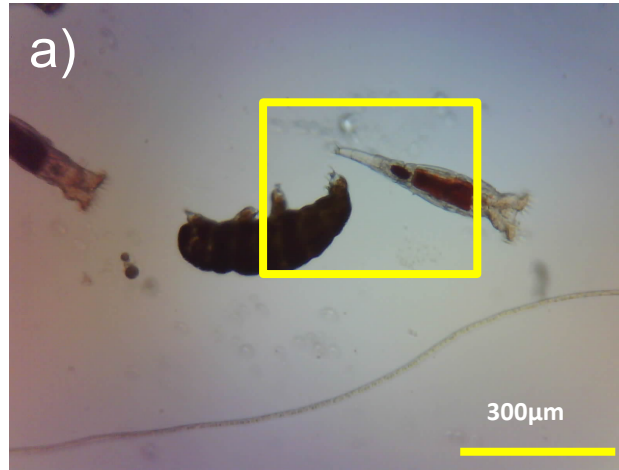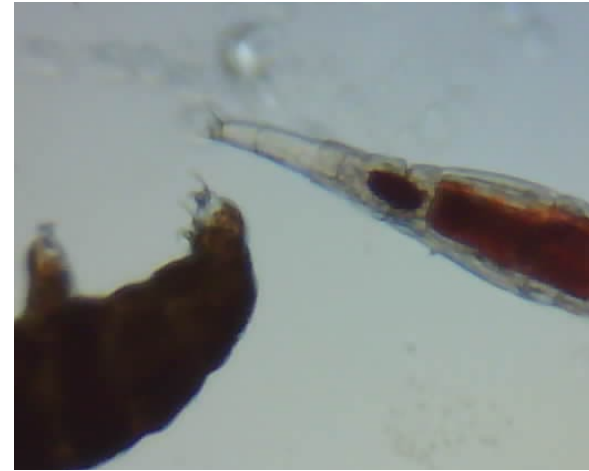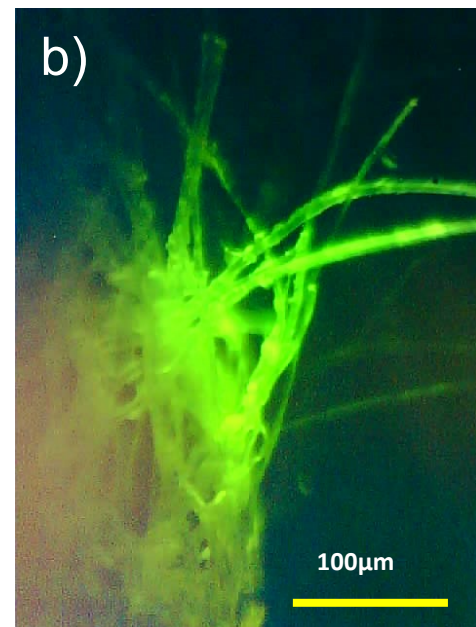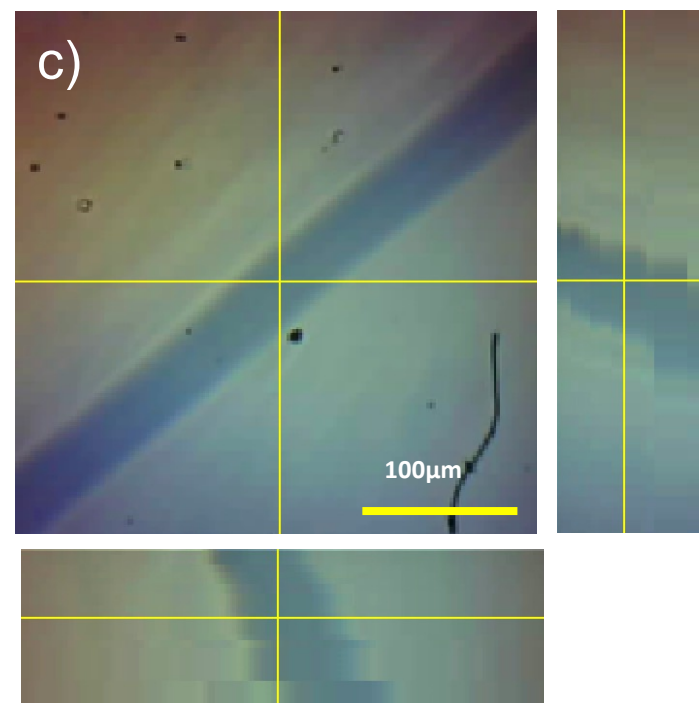

Supplement: S6 Fig — (a) Samples from a nearby pond. (b) Fluorescence capture using a blue laser pointer (450nm, laserlands.net) and a gel colour filter (LEE #13) on a yellow pen marker (Stabilo, Germany) on paper. (c) Focus stack of a line drawn by a marker on a coverglass on the surface of the Anglerfish microscope, showing cross-sections along z in x and y. (PDF) [file pone.0306654.s006.pdf]

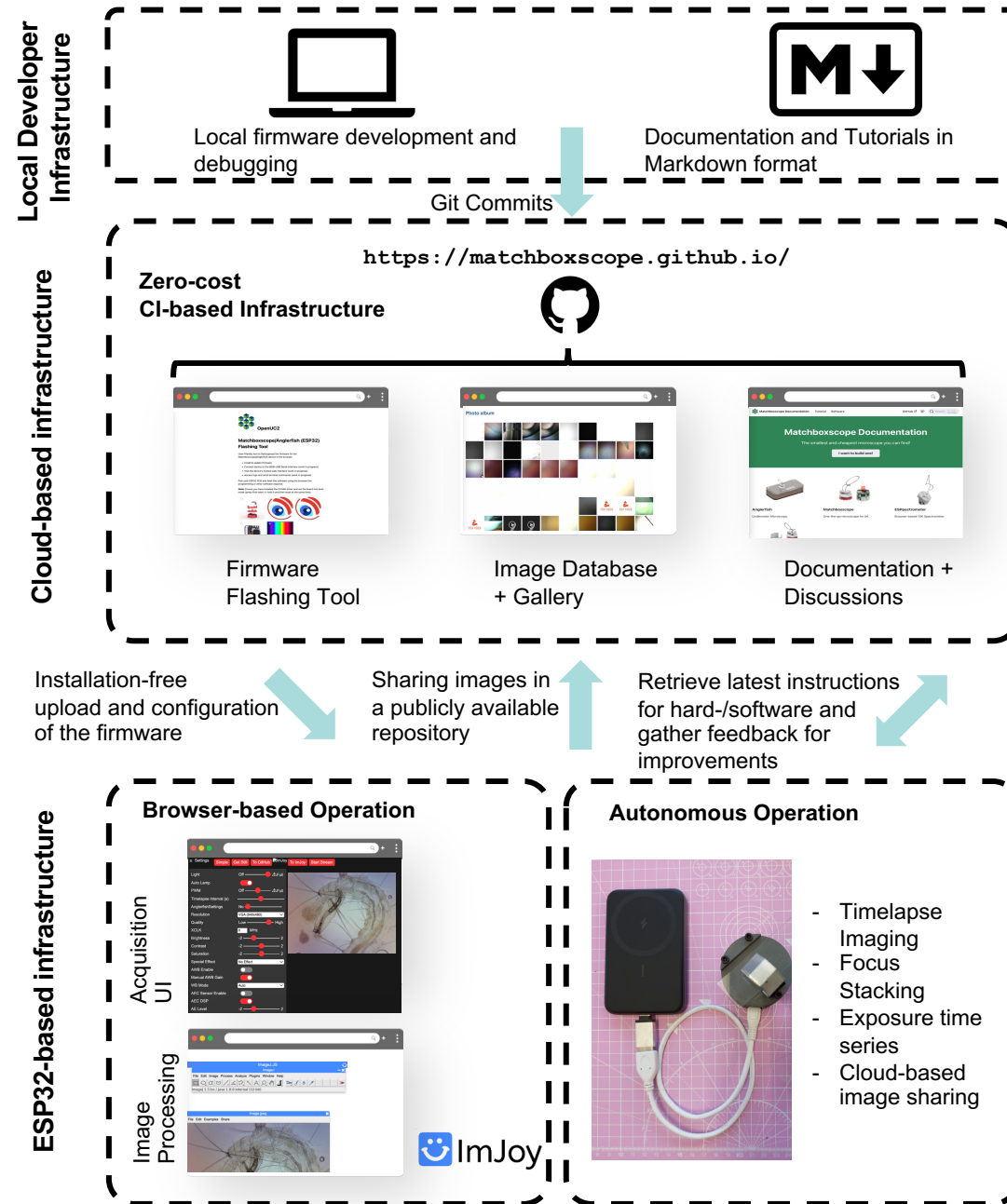

Supplement: S7 Fig — The pipeline is used to compile the firmware binaries, build the documentation website based on the open-source documentation building system Docusaurus, and host the image gallery of images uploaded from the ESPressoscope firmware. The firmware on the ESP32-CAM module runs in either interactive mode through a web browser or fully autonomous for timelapse imaging. (PDF) [file pone.0306654.s007.pdf]

Sharpness as a function of lens position in z-direction

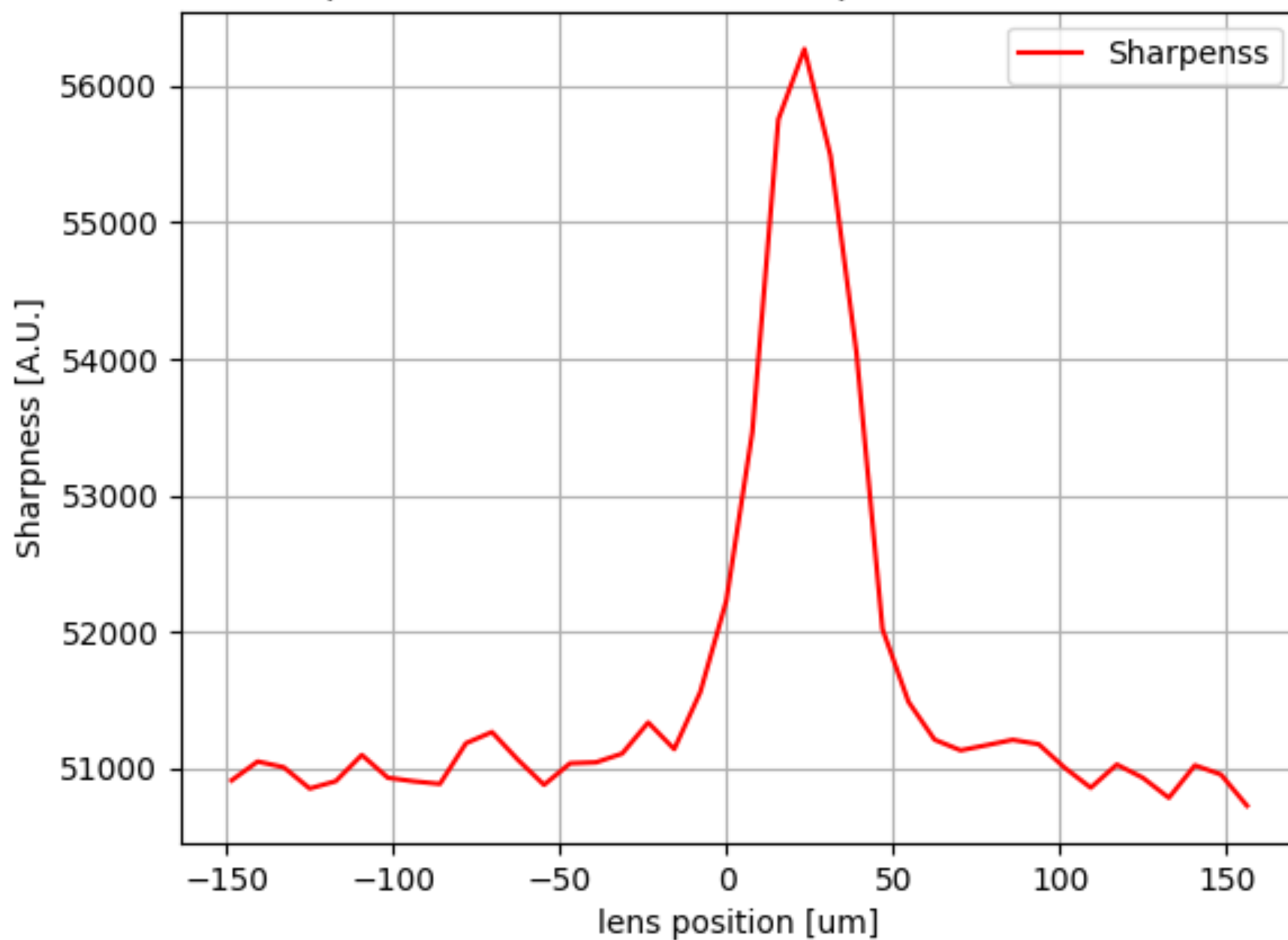

Supplement: S9 Fig — The graph measures the size of the JPEG stream that is decoded on the image to evaluate sharpness computationally. (PDF) [file pone.0306654.s009.pdf]
