# Supplementary material for: ESPressoscope: A small and powerful approach for in situ microscopy
Source: PLoS One. 2024 Oct 16;19(10):e0306654. doi: 10.1371/journal.pone.0306654 (PMC11482665; doi:10.1371/journal.pone.0306654)
Supplement: S3 Fig — A prepared slide from a Gossypium stem is used to illustrate how each illumination mechanism affects the illuminated field of view, visible contrast, homogeneity, and signal quality. Different mechanisms provide varying degrees of freedom, contrast, and usability, such as periscopic illumination, battery-driven LED, Toslink fiber-optic cable, Ikea USB lamp, and Neopixel LED matrix. (PDF) [file pone.0306654.s003.pdf]

	Periscope Illumination	Battery-driven LED	Toslink	LED LED Lamp	Neopixel Array
**Setup**	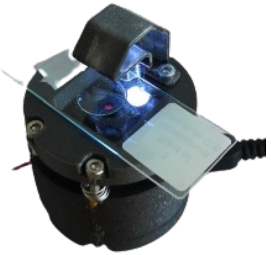	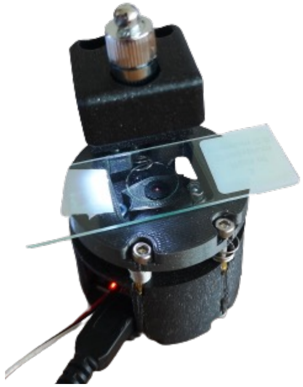	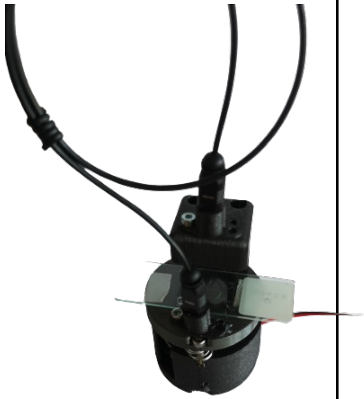	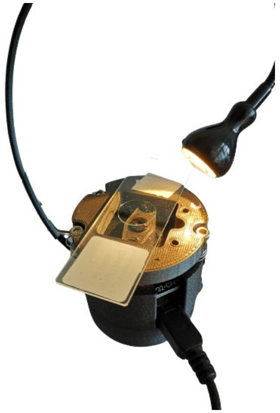	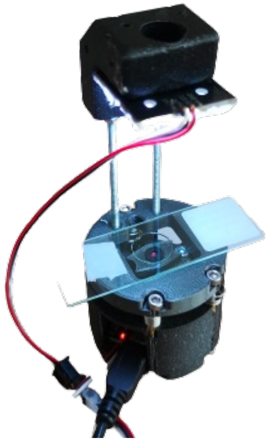
**Imaging Result** (Brightfield, optional: Darkfield)	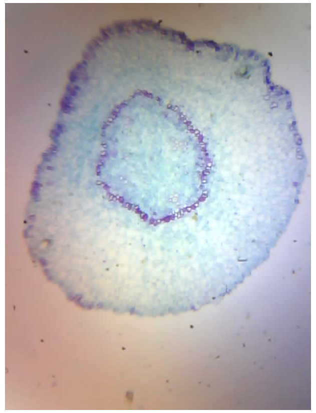	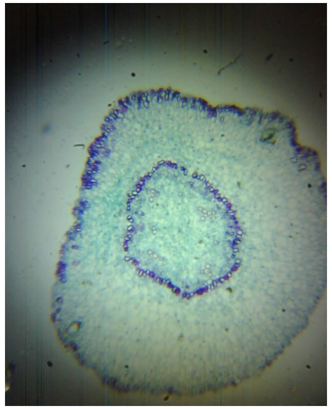 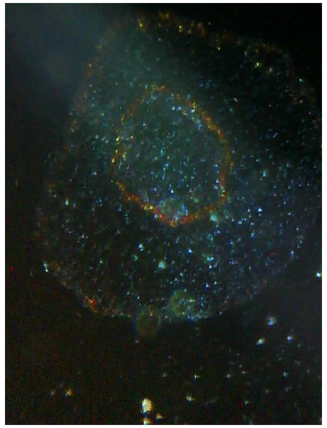	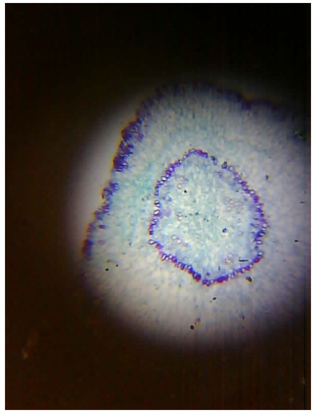	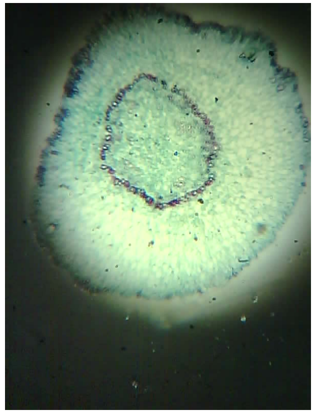 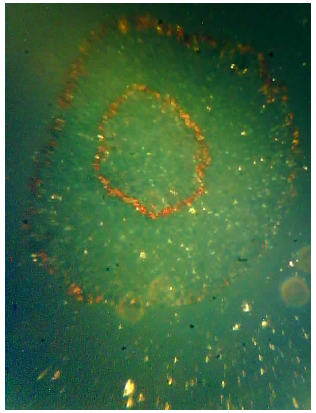	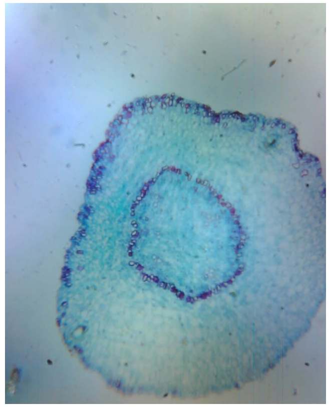
