# Supplementary material for: ESPressoscope: A small and powerful approach for in situ microscopy
Source: PLoS One. 2024 Oct 16;19(10):e0306654. doi: 10.1371/journal.pone.0306654 (PMC11482665; doi:10.1371/journal.pone.0306654)
Supplement: S8 Fig — Various configurations are created by combining an ESP32 camera module with additional components to realize specific functionalities. The ESPressoscope architecture is completely modular, allowing different add-ons to be used interchangeably with various configurations. Options listed in parentheses “(X)” represent possible alternatives to the recommended options. (PDF) [file pone.0306654.s008.pdf]

Name	Springloaded Focussing Stage	Magneti-loaded Focussing Stage	VCA Focusing Mechanism	Periscope Illumination	Lightguided Illumination	Ikea USB light	Neopixel Illumination	Waterproof LED lantern	Blue LED with 0.1 Printing Nozzle	Waterproof Container	Peristaltic Pump	Battery	Grating (1000 lines/mm)	3D printing nozzle (0.25mm)	Comment/ usecase	Compelxity (1=low, 3=high)	Approx. Time to build (min)	Est. Price
Matchboxscope	X	(X)		x				(x)							field work, only uses ESP32 + 3D printed components	1	15-20	10€
Incubatorscope	X	(X)	x				x	(x)				x			Focusstacking, Long-termseries on SD card	2	60-90	20€
Anglerfish	X		X		X			(x)		X		X			Autonomous In-Situ Imaging under water	3	60-120	30€
Fluidic-Scope	X					X		(x)			X				Field work, sampling of waterborn organisms	3	60-120	30€
ESPectrometer						X							X	X	Low-cost spectrometer to visualize wavelengths	2	30-60	20€
ESPHolo									x						Lensless imaging for inline hologrphy	1	15-20	10€
