# Supplementary material for: ESPressoscope: A small and powerful approach for in situ microscopy
Source: PLoS One. 2024 Oct 16;19(10):e0306654. doi: 10.1371/journal.pone.0306654 (PMC11482665; doi:10.1371/journal.pone.0306654)
Supplement: Attachment — Submitted filename: PlosOne Review_PONE-D-24-07702.pdf [file pone.0306654.s013.pdf]

Dear Editor,

Dear PLOS ONE Editorial Office,

I would like to thank you for your kind invitation to review the manuscript ID# PONE-D-24-07702 and entitled "ESPressoscope: a small and powerful platform for in situ microscopy".

Please find the review report below.

The article addresses the disadvantages of microscopy and proposes ESPressoscope, an alternative, inexpensive, and portable imaging system, and discusses microscopy solutions that enable new and more accessible experiments for biological and analytical applications. Please consider the comments below to improve the paper further:

1) From my perspective, the abstract section is well handled and conveys clear content about the article to the target readership.

2) The integration of firmware, user interface, and a browser-based graphical user interface (GUI) represents substantial contributions to both the paper and the broader scientific research within this field. These components not only enhance the functionality and usability of the proposed system but also pave the way for innovative advancements in the intersection of technology and scientific inquiry.

3) Introduction has provided some background research and highlighted their advantages and disadvantages.

   Regarding the Introduction section, it is recommended to include references for the content discussed in lines 42-48 to ensure transparency and provide readers with the necessary background information. This would enhance the credibility and academic rigor of the manuscript.

   "Other microscopes have been developed with the goal of making specific research techniques more accessible at high performance and low cost. One example is the 3D-printed and open-source OpenFlexure microscope, which is backed by a large user community (estimated 1k users) and is one of the first medically-certified microscopes used to identify malaria in blood smears . Another example is the Planktoscope,…."

4) Furthermore, it is suggested that the Introduction section be expanded to encompass a broader literature review. Currently, the review predominantly focuses on a limited number of handmade microscopes, while the title suggests a wider scope with the phrase '...platform for in situ microscopy'. In the existing literature, there have been documented instances of miniature imagers constructed from webcams or low-cost sensors, offering convenient assembly and cost-effective in situ imaging of biological structures. Therefore, it is advisable for the authors to consider evaluating these systems to enhance the comprehensiveness of their study.

   It is recommended that the following articles be assessed for their relevance to similar studies, as an example:

   Tseng, D., Mudanyali, O., Oztoprak, C., Isikman, S. O., Sencan, I., Yaglidere, O., & Ozcan, A. (2010). Lensfree microscopy on a cellphone. *Lab on a Chip*, *10*(14), 1787-1792.

   Polat, A., Hassan, S., Yildirim, I., Oliver, L. E., Mostafaei, M., Kumar, S., ... & Zhang, Y. S. (2019). A miniaturized optical tomography platform for volumetric imaging of engineered living systems. *Lab on a Chip*, *19*(4), 550-561.

   Greenbaum, A., Luo, W., Su, T. W., Göröcs, Z., Xue, L., Isikman, S. O., ... & Ozcan, A. (2012). Imaging without lenses: achievements and remaining challenges of wide-field on-chip microscopy. *Nature methods*, *9*(9), 889-895.

   Kim, S. B., Bae, H., Cha, J. M., Moon, S. J., Dokmeci, M. R., Cropek, D. M., & Khademhosseini, A. (2011). A cell-based biosensor for real-time detection of cardiotoxicity using lensfree imaging. *Lab on a Chip*, *11*(10), 1801-1807.

   Zhu, H., Yaglidere, O., Su, T. W., Tseng, D., & Ozcan, A. (2011). Cost-effective and compact wide-field fluorescent imaging on a cell-phone. *Lab on a Chip*, *11*(2), 315-322.

   Polat, A., & Göktürk, D. (2022). An alternative approach to tracing the volumic proliferation development of an entire tumor spheroid in 3D through a mini-Opto tomography platform. *Micron*, *152*, 103173.

5) I found Figure 1 to be lacking in descriptiveness from the reader's perspective. It wasn't clear to me whether Matchboxscope, Anglerfish, ESPlanktoscope, ESPectrophotometer, and HoloESP in Figure 1 represent components of the ESPressoscope platform, or if the ESPressoscope platform transforms into a different model depending on their integration. Clarification on this aspect would enhance understanding. The caption is also recommended to be revised accordingly.

6) In lines (133-135), there is some complexity regarding the variables used. It would be beneficial to clarify the meaning of '**d**' and whether '≈ 4mm' also refers to '**a'**'. Additionally, further explanation is needed on how the effective pixel size was calculated.

An image on the sensor is focused and magnified if a < a_0. However, to create a compact device with the ESP32-CAM board's d 4 mm sensor and f' 4 mm focal length of the objective, we have chosen a' ≈ 18mm and ≈ 4mm, leading to a total magnification of 4 and an effective pixel size of ≈ 0.6µm.

7) Similarly, what is 0.23 and 1.2 µm in line 137?

8) In Fig 2 caption, there should be the explanation of a). Additionally, how the graph in Fig 2c created? I think it is not USF Chart Group of 6,7; it is 1D profile of USF chart…

9) If I am not mistaken, I could not find the supplementary Figures in the file.

10) Is *supplement Figure 2* Figure S2? Is Figure S1 *supplement Figure 1*? I recommend using Figure S1, Figure S2... by eliminating these confusions and following a standardization throughout the article.

11) There is no a) in Figure 5. The all captions should be reviewed and revised.

12) The manuscript adeptly elucidates the conclusion, limitations, and avenues for future work. The conclusion effectively summarizes key findings while the discussion of limitations provides valuable insights into the study's scope and potential areas for improvement. Furthermore, the delineation of future work highlights the authors' forward-thinking approach, setting the stage for continued advancements in the field.

13) I would like to offer a humble suggestion to the authors to conduct a thorough review of the entire article, focusing on eliminating minor errors and revising any parts that may be difficult to understand. Additionally, I recommend revisiting the flow of the subject matter where necessary, reassessing the priorities in the sequence of Figure descriptions within the text to enhance coherence, and reviewing the Figure captions for clarity and accuracy.

I wish you success in implementing the suggested improvements.
