# Supplementary material for: ESPressoscope: A small and powerful approach for in situ microscopy
Source: PLoS One. 2024 Oct 16;19(10):e0306654. doi: 10.1371/journal.pone.0306654 (PMC11482665; doi:10.1371/journal.pone.0306654)
Supplement: Attachment — Submitted filename: Reviewer 2.docx [file pone.0306654.s014.docx]

The manuscript proposes a novel and efficient method utilizing a simple microcontroller board, not only as a portable and field-ready digital microscope but also as a versatile platform adaptable to various imaging applications. I believe the manuscript is well-written and merits publication. However, there are two minor revisions needed. Firstly, the quality of figures is low and does not meet standard requirements. Secondly, while the "Conclusions and Outlook" section discusses the limitations and drawbacks of the method, adding experiments and analyzing reasons for the proposed method's poor performance in some cases can enhance the manuscript's value. This addition is highly recommended.
